# Governing AI for Pharmacovigilance in Low-Income Countries: Systems Perspective

**DOI:** 10.2196/96374

**Published:** 2026-09-28

**Authors:** Garang Majok Dut

**Affiliations:** 1 International Centre for Future Health Systems (ICFHS), UNSW Medicine & Health, UNSW Sydney, Level 5, Health Translation Building, Sydney, New South Wales, 2052, Australia, 61 293851000; 2 Therapeutic Goods Administration (TGA), Department of Health, Disability and Ageing, Australian Government, Canberra, Australian Capital Territory, Australia

**Keywords:** pharmacovigilance, artificial intelligence, pharmaceutical governance, Programme for International Drug Monitoring, VigiAccess, substandard medicines, adverse drug reactions, adverse drug events, South Sudan, Australia

## Abstract

Gaps in pharmaceutical governance could widen with the adoption of AI, even as AI promises better pharmacovigilance in low-income countries (LICs). While advanced regulatory systems like Australia’s are integrating AI into pharmaceutical governance, LICs with underdeveloped regulatory capabilities, such as South Sudan, lag behind. The potential divergence disorients the World Health Organization’s “Medicine Without Harm” agenda and effective global pharmacovigilance. Moreover, evolving global governance initiatives, including the newly established United Nations scientific panel on AI, may be hampered by this global divergence in capabilities. This makes 3 critical interrelated questions: what are the moral trade-offs in the introduction of AI in health care, what power dynamics impact the introduction of AI into health systems, and how could AI be used for pharmacovigilance in LICs? This viewpoint aims at informing global policies and regulations on AI in pharmacovigilance. It uses clinical, policy, and regulatory practitioner insights to synthesize evidence on the ethical, economic, and clinical contours of AI in pharmacovigilance. It contrasts the high-income context of Australia with the low-income context of South Sudan and shows that national capabilities are instrumental for institutionalizing global practice. It identifies current ethical challenges with applying AI and digital health, which straddle epistemic, normative, and metaethical domains, such as misguidance, cultural devaluation, and trust deficit. These filter into demerits observed with current applications of AI to pharmacovigilance, from the detection of adverse drug events and adverse drug reactions to the simulation of clinical trials. The merits of current applications are multiple and depend on data quality, ranging from the detection of adverse drug reactions to real-time surveillance of medical errors and predictive application to population risk quantification of adverse drug events. The widening gaps in global capabilities amid rapid evolution of AI suggest the need for inclusive global governance in the early stages, especially because AI may be deterministic and effects may not be retrospectively surmountable. The viewpoint also assesses the sufficiency of current evaluation frameworks, noting that health economic models currently lag in capturing gains and losses from the adoption of AI in health systems, digital health frameworks are largely retrospective and overlook sociopolitical and financial contexts, and influential service-oriented frameworks for health systems overlook outcomes. It observes that, although AI could be harnessed across the breadth of the pharmaceutical system, effective evaluation of potential risks is hampered by upstream decisions in software development and procurement, which preclude aspects of subsequent application. This introduces inscrutability and weakens clinicians’ role in risk adjudication, which may worsen with nonrepresentative evolution of AI. Using these insights and a case study on the low-income context of South Sudan, the viewpoint commends an integrated health systems framework and country-level investments in infrastructure and regulatory capabilities as requisites for effective global governance and equitable use of AI in pharmacovigilance.

## Background

Regulatory systems ensure safety, quality, and effectiveness of pharmaceuticals [1]. In low-income countries (LICs), underdeveloped regulatory capabilities contribute to substandard, falsified, unregistered, or unlicensed medicines [2]. This could worsen if advances in AI outpace regulatory systems [3]. AI refers to computer systems that are devised to think or act rationally, like humans [4]. Similarly, mobile health regards the application of mobile or wearable devices in digital health services [5]. By using currently available simple and cheap technologies, like mobile phones, AI and mobile health enable process automation [6], pattern recognition [7], and decision-making with capacity to learn from large datasets [8].

AI is leveraged in drug discovery [9,10] and is increasingly applied in pharmaceutical regulation [11,12]. These applications include “pharmacovigilance,” which aims at “detection, assessment, comprehension and prevention of medicines-related problems” [13,14]. The World Health Organization (WHO), for instance, deployed AI during the COVID-19 pandemic to monitor adverse reactions to COVID-19 vaccines [15]. The WHO has also integrated it into version 2.0 of the Epidemic Intelligence from Open Sources system [16]. Process automation and predictive analytics enhance signal detection of adverse drug events (ADEs) or adverse drug reactions (ADRs), predict drug side effects, streamline safety reporting, map drug-drug interactions, delimit the population toxicity profile for a drug, and simulate clinical trials [17]. By early 2020, China harnessed AI in the pandemic response [18], and France used it for pharmacovigilance in 2021 [19]. Currently, the United States Food and Drug Administration is integrating AI into scientific reviews for pharmaceuticals [20]. Although these applications have positive externalities for LICs, including approval for drugs on the WHO’s Essential Medicines List [21], their potential impact in LICs is underexamined.

Pharmacovigilance in LICs is constrained by fiscal limitations, skills gaps, and poorly integrated regulations [22]. This limits access to high-quality medicines, which hampers health care effectiveness in LICs such as South Sudan [23,24]. Emerging evidence suggests AI could improve signal intelligence and, therefore, regulatory enforcement [17]. However, signal detection for ADEs is undermined by poor data integration and voluntary reporting, even in rich settings such as Australia, where compulsory reports by medicines sponsors outweigh the 16% voluntary reports by clinicians [25]. Automated reporting with AI could circumvent this constraint, as demonstrated in symmetry analyses of large health datasets [26-28]. Nonetheless, the evolving regulatory landscape—including whether AI should be regulated as a medical device [29,30]—risks neglecting LICs as the capabilities gap widens.

The current regulatory vacuum compounds underrepresentation in clinical trials [31,32], medicines production [33], and the dominance of influential global actors in determining the WHO’s Essential Medicines List [21,34]. Few older adults, Black people, children, women, and Indigenous people participate in clinical trials [35]. Barriers in LICs include limited financial capital, weak regulatory and ethical governance, and an underdeveloped research ecosystem [36]. However, AI’s impact on these inequities could be moderated by early interventions, including in digital twins [37,38] and diverse genetic databanks for training large language models (LLMs) [39]. These remain unexplored from a systems perspective.

Remedies for global inequity in pharmaceuticals include purchase agreements [40,41], which have varied effects [42,43], and in-kind foreign aid or humanitarian supplies [44], which are suboptimal [45,46]. However, pharmacovigilance is less mitigated by these measures: Africa and Asia struggle with controlling substandard or counterfeit medicines [47,48]. External regulatory support comes in 3 forms: direct agreements between LICs and regulators such as the European Medicines Agency, reliance on regulatory standards and methods of advanced economies, and research and capacity-building support from established regulators [49]. In the European Union, these outward measures are informed by its agenda on universal health coverage [50]. European Medicines Agency advises on medicines destined for third countries [51]; the European Council’s Directorate General for Research and Innovation finances development and testing of essential medicines [52]; and the EU requires compliance with WHO quality assurance standards [53]. The effects of these measures are mixed for Africa [52], and inequities may widen with divergent AI regulations [54,55]. Albeit peripheral to pharmacovigilance, a recently established 40-member UN scientific panel on AI [56] promises global coordination.

WHO’s “Medication Without Harm” agenda aims at stemming harm from medicines [57]. This builds on its commitment to pharmacovigilance, heralded by thalidomide in 1961 and later broadened to traditional and herbal medicines, complementary medicines, blood products, and medical devices [58]. The latest guidance emphasizes iatrogenic harm and commends national strategies for public and patient engagement, medicines monitoring, upskilling of health professionals, and establishment of systems for medicines management [57]. These are suitably adapted in rich settings, with the Australian Commission on Safety and Quality in Health Care, for instance, striving to mitigate “medication errors, ADEs and medication-related harm” [59]. Australia’s national strategy informs clinical governance for curbing polypharmacy, mitigating harm from high-risk medicines, and enhancing communication for safe medicines use [59]. Since 1978, the WHO has supported global pharmacovigilance through Sweden’s capabilities in registry and pharmacoepidemiologic methods at the Uppsala Monitoring Center [58]. By July 2023, the Uppsala Monitoring Center–managed global database—“VigiBase”—had received 35 million Individual Case Safety Reports under the WHO Programme for International Drug Monitoring (PIDM), which was established in 1968 [60]. Adding “VigiAccess” to this capability in April 2015 sought to leverage the digital revolution [61]. Underdeveloped capabilities in LICs constrain these efforts.

This viewpoint aims at informing global policy and regulatory practice on AI in pharmacovigilance. It uses clinical, policy, and regulatory practitioner insights to synthesize evidence on ethical, economic, and clinical contours of AI in pharmacovigilance and addresses 3 interrelated questions: what are the moral trade-offs in the introduction of AI in health care, what power dynamics impact the introduction of AI into health systems, and how could AI be used for pharmacovigilance in LICs? The analysis benchmarks Australia and South Sudan as respective examples of advanced and weak regulatory systems, using the WHO’s template for a national pharmacovigilance system. While not generalizable across LICs, this binary comparison of both extremes exposes the chasm that should be bridged in regulatory capabilities for equitable and effective global governance of AI in pharmacovigilance. It considers how AI is getting integrated in Australia’s advanced system, while delineating the constraints and opportunities for LICs through a case study on South Sudan. Moreover, a schema of AI-supported pharmacovigilance systems centers cybersecurity and risk management to underscore the critical role of humans in the loop. Governance challenges are further expounded through critical appraisal of ethical concerns and current applications of AI in pharmacovigilance, mapping these to major themes in the literature. Evaluative frameworks were then compared with a view to moderating these challenges, and conclusions subsequently drawn from a systems perspective. It argues that, although AI is potentially transformative, limited regulatory capacity in LICs constrains pharmacovigilance, contributes to poor health outcomes through substandard medicines, and could worsen global inequity with the adoption of AI. Equitable deployment of AI would benefit from integrated health systems evaluation and infrastructure and regulatory improvements in LICs.

The viewpoint progresses as follows: (1) it delimits WHO’s commendation for a pharmacovigilance system and contrasts its domestication in the rich context of Australia with the low-income context of South Sudan; (2) it examines implications of limited pharmacovigilance in LICs; (3) it considers how AI could redress these challenges; (4) digital health and health systems evaluation frameworks are interrogated in their capacity to mitigate harm from AI; and (5) a case study on South Sudan demonstrates opportunities and constraints in LICs.

## A National Pharmacovigilance System Is a Function of Domestic Capabilities

WHO recommendations for a pharmacovigilance system stem from consultations among stakeholders, including WHO, Gavi Alliance, expert panels, and national governments [62]. These evolved, under the aegis of the WHO Advisory Committee on the Safety of Medicinal Products, into the minimum requirements encompassing 5 domains: a national pharmacovigilance center, a national spontaneous reporting system, a national database, an advisory committee, and a communication strategy (Figure 1). These integrate to achieve 8 primary goals: promoting pharmacovigilance, signal detection, risk assessment and management, quality control, risk communication, provision of public information, maintenance of drug use information, and identifying unregulated prescription [13,62].

A pharmacovigilance system establishes functionalities around what to report, when to report, how to report, and how to action reports on ADEs or ADRs [14,62]. These are embodied within medicines regulators, such as Australia’s Therapeutic Goods Administration, United States Food and Drug Administration, and South Sudan’s Drug and Food Control Authority (DFCA). Medicines regulators use reviews of Pharmaceutical Risk Assessment Committee reports to update safety warnings, inform evaluations for market authorization, and/or prompt recalls of regulated products [13]. AI promises transformation where tasks involve pattern recognition, but operationalization and validation within existing systems remain challenging [63,64]. Although digitalization may allow for risk assessment, resource gaps in LICs could compound existing challenges.

In rich countries, the WHO’s commendations are readily distilled into competencies. In Australia, for instance, these have evolved into practice-oriented guidelines (Figure 2). By contrast, small-sized and resource-constrained countries must prioritize and leverage scale through regionalization and digital technology [1]. Experience in South Sudan shows limited skills transfer with e-learning platforms for pharmaceutical management [65], suggesting challenges with complex capabilities.

Unlike Australia (Figure 2), weak institutions, poor infrastructure, and dominance of external actors constrain regulatory capacity in South Sudan [66]. Evidently, the majority of signals actioned by DFCA originated from outside its laboratories (Figure 3). This incapacitation is prevalent across LICs [67] and often assuaged with regional capabilities, such as East African Regulatory Affairs Professionals Association (Figure 3) or PIDM (Figure 1). However, amid divergent regulatory preferences [55,68], the likely impact of AI on this regulatory support system remains unexplored.

**Figure 1.**
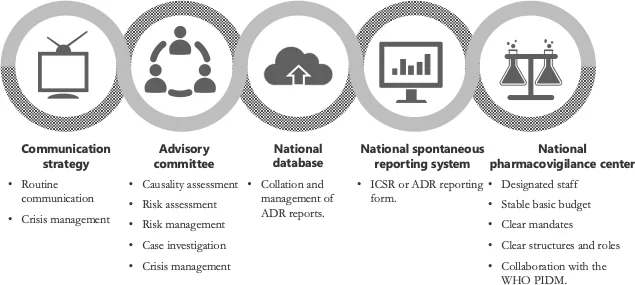
Minimum composition of national pharmacovigilance system. ADR: adverse drug reaction; ICSR: individual case safety report; PIDM: Programme for International Drug Monitoring; WHO: World Health Organization. Source: author, based on WHO [62].

**Figure 2.**
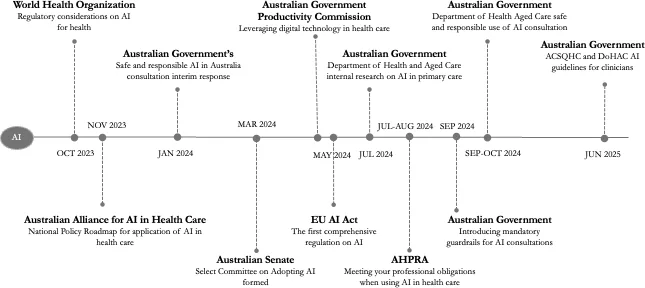
Australia’s regulatory and policy response to AI in health care benefited from robust domestic capabilities. ACSQHC: Australian Commission on Safety and Quality in Health Care; AHPRA: Australian Health Practitioner Regulation Agency; DoHAC: Department of Health and Aged Care. Source: author’s compilation.

**Figure 3.**
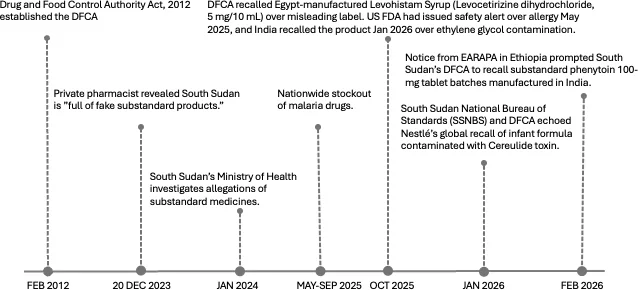
Pharmaceutical regulation in South Sudan is constrained by limited domestic capabilities. DFCA: Drug and Food Control Authority; EARAPA: East African Regulatory Affairs Professionals Association; US FDA: United States Food and Drug Administration. Source: author’s compilation.

## Weak Pharmaceutical Regulatory Systems Undergird Poor Quality Medicines

Poor-quality medicines contribute to the scourge of infectious diseases [69-72] and noncommunicable diseases [73,74]. These are estimated at 35% for falsified antimalarials in Africa [75], 70% for counterfeit drugs in Africa or Asia [48], and 88.4% for substandard antimalarials in Africa—relative to 53% for substandard antimalarials in Southeast Asia [47]. This explains 12,300 malaria-related annual deaths in Nigeria [76] and 8.1% annual excess deaths among Zambian children [72]. Globally, 1 million deaths result from counterfeit medicines, including 200,000 deaths due to fake antimalarials in Africa [48]. Besides health impacts, these impose economic and social costs [77,78], including US $150 million annually in Kinshasa and Katanga regions of the Democratic Republic of the Congo [79], US $193 million in Benin [80], US $31 million in Uganda [81], and US $893 million in Nigeria [76]. This pattern is driven by unaffordable costs of authorized medicines, weak medicines regulations and enforcement, and corruption [82]. They disproportionately impact the poorest wealth quintile [83], and culminate in ineffective treatment, distrust in therapeutics, and curtailed pharmaceutical investments amid competition with counterfeits [46,48].

The nomenclature that describes this scourge—substandard/spurious/falsified/falsely-labeled/counterfeit drugs [48]—doesn’t convey causes of poor quality [84]. However, concern about public harm is implied in “falsified medicine,” while “counterfeit medicine” highlights negation of intellectual property, and “fake medicine” suggests defectiveness [85]. Moreover, “substandard” drugs reflect deviation from specification, while “unregistered or unlicensed” denotes nonapproval by the regulator [67,85]. The significance of nomenclature gains as digitalization of pharmacies amplifies risks [48]. Although evidence is scarce, firmer intellectual property rights enable pharmaceutical monopolies, which may lessen medicines availability and affordability [86].

Brand competition and price adjustment improve affordability of patented drugs [87]. Between 2001 and 2016, the Trade-Related Aspects of Intellectual Property Rights agreement and Public Health improved drug availability in 176 instances in 89 countries, among which 84% covered 14 different conditions [88]. Fluidity in global preferences and definitions explains variations in estimates and regulation of counterfeit medicines [89]. The estimated 10% of ADRs in the WHO global surveillance system obscures 90% of instances of drug ineffectiveness, due to low dose or absence of active ingredient [48], or 94% median rate of underreporting in pharmacovigilance systems [90]. Nonetheless, harm from falsified medicines renders other categories suspect with public harm [47,48,72,75].

Regulations are crucial for good health outcomes, brand integrity, and health-enhancing innovations [47,48,91]. The estimated US $75 to US $200 billion market for counterfeit drugs [92] suggests profit motives that compound limited visibility and strong links to China, India, and Russia, and confound legitimate medicines exports [48,84,93]. Digital methods for curbing fake medicines include use of mobile, radio frequency identification, online verification, blockchain technology, and advanced computation methods [94]. However, 50% of drugs sold over the internet are falsified or counterfeit, which undermines digitalization [48]. Moreover, difficulty with visual distinction between drugs limits digital methods and underscores a role for field methods and advanced laboratory methods [89]. Bolstering these regulatory capabilities demands global cooperation and investments [95].

## The AI Dividend Depends on Domestic Infrastructure and Regulatory Capabilities

Current applications of AI are constrained by data quantity and quality [90], but well-trained LLMs could detect ADEs and ADRs potentially missed by a professional or predict occurrence of ADR and/or rank it on a severity scale [96-103]. Digital twinning also enables simulation of clinical trials [37] and could improve trial representativeness and lessen inequity in outcomes and pharmacogenomics [36,104]. These applications, although currently aspirational in use of mobile devices and requiring future research into generalizability, could leverage cheap technologies to complement human capabilities (Figure 4). These potential gains are discernible from a health economics perspective [105]. Wi-Fi and videoconferencing enable digital health care [106,107], which is increasingly feasible in South Sudan [23] and amenable to LLMs [108,109]. Gains could also proceed from data-driven needs assessment and demand-driven financing of pharmaceuticals (Figure 5), which is underdeveloped in South Sudan [24].

These could be further enhanced with policy and regulatory interventions: in Australia, for instance, literacy and technical know-how influence individuals’ access, utilization, and participation in agenda-setting for digital health care [110-112]. The poor, digitally illiterate, or less-educated populations were excluded from related debates, and access gaps emerged along rurality, age, and income categories [110,113]. In the UK, data sharing between Google’s DeepMind Technologies Limited and National Health Service stumbled on privacy and ethical concerns [103]. These could filter into AI, so gaps in current understanding impoverish full appraisal of AI’s value [114]. These could also be pronounced across cultural groups, due to nonrepresentative data (Table 1).

In medicine, knowledge vests doctors with greater power, relative to patients, and professional training in medical law and ethics redresses this imbalance [115]. However, using AI as a decision aid means some decisions are preloaded into software and, therefore, into the economics of health budgets [116], resulting in path dependence or AI determinism. Consequently, policymakers or administrators involved in purchase or health technology assessment, and programmers, become complicit in ethics that obtain [117,118]. The adoption and utilization are also impacted by stakeholder interests within a health system [119]. Moreover, AI could distort the doctor-patient relationship, including anchoring attention away from the patient [120,121]. The resultant physical or psychological barrier could discount empathic care [122]. Furthermore, technology companies are dominant actors in AI and could depersonalize ethics as “virtual sovereignty” obtains [123]. This invokes irony because AI promises advances in personalized medicine [124]. The potential inscrutability and limited traceability with AI introduces a permanency that could amplify on scale and across cultures and jurisdictions [125-132]. Similarly, cultural debasement with the unrepresentative evolution of AI [118,120] undermines the global vision of AI governance (Table 1).

**Figure 4.**
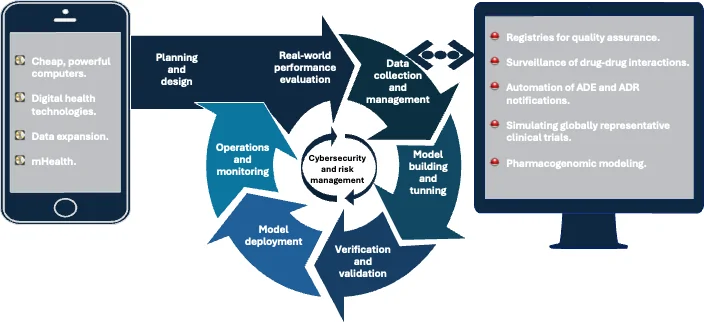
AI-supported pharmacovigilance system could leverage large datasets for human-interfaced surveillance. ADE: adverse drug event; ADR: adverse drug reaction; mHealth: mobile health. Source: author’s conceptualization.

**Figure 5.**
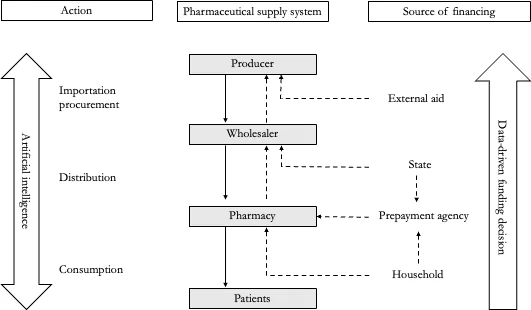
AI could embed pharmacovigilance into financing of pharmaceuticals and logistics, management, and information systems. Source: author’s conceptualization.

**Table 1.** Ethical considerations in AI and digital health necessitate upstream interventions.

Ethical concerns	Explanation	Potential outcome
Epistemic ethics [125-132]		
Inconclusiveness	Algorithms are probabilities that are insufficient for a causal relationship.	Inappropriate calibration could result in misplaced diagnosis in patients.
Inscrutability	Limited oversight of the data used to train the algorithm or used in decision-making.	A clinical decision support system may result in overprescribing or underprescribing without clarity about the basis of the decision.
Misguidance	Conclusions are only as robust as the data fed into the algorithm.	If an image bank used to train the algorithm has inherent bias, such as on the basis of ethnicity, its results may be persistently biased.
Exclusivity	Dominant firms and data sources may entrench regionally specific assumptions within algorithms (eg, Western individualism vs Ubuntu).	Cultural dispossession and systemic inequity.
Normative ethics [125-127,129-132]		
Unfair outcomes	An action may prove to have an impact on one group of people.	Minority groups may be discriminated against by an algorithm that learns to normalize or prioritize patterns evident in the majority.
Transformative effects	Profiling, which is intrinsic to algorithms, reconceptualizes reality in unanticipated ways.	Passive data from personal devices may filter into algorithms, which in turn impact recommendations for an individual, who in both instances has limited oversight.
Cultural devaluation	Discount for community cohesion in pursuit of individual autonomy.	AI likely to aggravate community disempowerment and power dynamics between the Global North and Africa.
Metaethics [125,127,129,131,132]		
Traceability	Hard to debug algorithmic errors and assign responsibility for the harm caused.	Negative outcomes pursuant to errors from decision aid software do not present a clear chain of responsibility or means for preventing future harm.
Debased governance	Platforms are being developed by technology giants, without expected duty of care.	Self-regulating capacity of the health care profession is challenged by the power of a nonbinding entity that does not share in trust vested in the profession by society and the duty owed to society by the profession.
Trust deficit	Vulnerable to hacking and patient-doctor relationship harmed by diminishing doctors’ control over computer-based algorithms.	Greater danger of harm to society if patients trust computer algorithms more than their doctors; decaying trust in the health system.

## AI in Pharmacovigilance Requires Digitalization and Quality Databases

Application of AI in pharmacovigilance encompasses data processing for detection of ADEs and ADRs (57.6%), classification of safety reports (21.2%), extraction of drug-drug reactions (7.6%) or population-based toxicity analysis (7.6%), side-effect projections (3.0%), clinical trial modeling (1.5%), and controlling for uncertainties in diagnostic classifications (1.5%) [17]. Moreover, social media is increasingly mined for ADRs and ADEs and currently limited by data quality [133-135]. Similarly, drug-drug interaction, which increases with polypharmacy, could be mitigated with AI predictions [136,137]. Premarket application could also build a database of side effects [138]. However, due to the complexity of medical text, natural language processing is currently inferior to manual review [139].

Pharmaceutical regulation in South Sudan lags in these capabilities. It is premised on the DFCA Act, 2012, which established the Pharmaceutical Quality Control Laboratory (Chapter X) for quality assurance and disposal of regulated products [140]. However, its legislated functions remain underdeveloped, resulting in DFCA largely enforcing signals from outside its designated laboratory (Figure 3). This impresses DFCA as incapacitated in requisite capabilities, except for communication system or strategy (Figure 1). The varied ways of harnessing AI in pharmacovigilance—including detection of ADEs and ADRs; processing safety reports; extraction of drug-drug interactions; drug toxicity modeling for personalized care; predicting side effects; simulating clinical trials; and diagnostics (Table 2)—could be improved with representative databases and digitalization in LICs. Current divergence in global capabilities and fractured regulatory regime [55,68] call for recalibration of “good AI society” [68].

**Table 2.** Applications of AI in pharmacovigilance are varied and limited by data quality.

Element of pharmacovigilance	Merits	Demerits
Detection of ADEs^a^ and ADRs^b^ [96,97,133-136,141-150]	Detection of ADRs, which may often be missed by medical professionals. Concurrent evaluation of large datasets. Leverages publicly available online information. Mitigation of ADE from polypharmacy. Quality assurance through prediction of ADEs and prompts for ADE reports. Detection of hypoglycemic incidents (eg, HypoDetect) to tailor treatment.	Excess noise within data due to irregular text. Quality trade-off between manual screening for low-level social media and natural language processing for higher levels of social media data.
Processing safety reports [151,152]	Useful for screening and mining large, unstructured or semistructured datasets on adverse events and near-miss reports on passive surveillance systems. Identification of allergic reactions in free-text narratives of hospital safety reports and evaluation of generalization. Real-time event surveillance of medical errors. Safety-net system for early detection of adverse events with likelihood of severe harm or death.	Inferior to manual review in analyzing technically complex medical texts. Limited by digitization of clinical records.
Extraction of drug-drug interactions [153-155]	Enables extraction of drug-drug interactions. Enables prediction of drug-drug interactions. Useful for drug safety monitoring in clinical settings. Harnesses laboratory and treatment data to detect ADEs resulting from drug-drug interactions. Predictive capacity for drug-drug interactions on the basis of a small cluster of data.	Varied methods (eg, kernel-based and feature-based) applied and not yet harmonized across laboratory, treatment, and population datasets.
Drug toxicity or guidance for personalized care [136,156,157]	Helps identify population risk categories for ADEs. Could personalize care by matching propensity scores to population-based risk categories. Helps target therapy by mapping pharmacogenomic susceptibility.	Risk of overfitting predictive models.
Prediction of side effects [158,159]	Predicts side effects from drugs. Supports literature-enhanced postmarket surveillance. Supports tumor biomarker-based risk prediction for ADRs. Future-ready for mechanism-oriented ADR research.	Dependent on data quality, evidence in literature, and reliability of biomarkers.
Simulation of clinical trials [160-162]	Simulation of clinical trials and adverse events through integration of real-world data. High sensitivity of simulation to reflecting clinical trial risk ratios of serious adverse events.	Depends on reliable and representative real-world data.
Integrated prediction of uncertainties and diagnostic classification [163,164]	Integrates predicted uncertainties into computer-aided diagnostics for patient safety. Complements determination of drug safety profile.	Scarcity of studies adapting the uncertainty quantification to physiological markers.

a ADEs: adverse drug events.

b ADRs: adverse drug reactions.

## Evaluative Frameworks Should Beware AI Determinism

Evaluation frameworks are currently limited in appraising all contours of AI [165]. Health economic models are outpaced [166], and this compounds limitations with digital health frameworks. “Benefits Management Framework,” for instance, is retrospective [167] and could overlook negative externalities and sociopolitical factors. “Digital Maturity Evaluation Framework” [168] ignores health financing and other levers. Moreover, “Nonadoption, Abandonment, Scale-up, Spread, and Sustainability Framework” [169] is service-oriented and neglects outcomes. Individually, each of the digital health evaluation frameworks is inadequate for capturing the full spectrum of technology investment evaluation, financing, adoption, and impact evaluation within health systems (Table 3).

**Table 3.** Digital health evaluation frameworks overlook contours of AI.

Framework	Digital Maturity Evaluation Framework [167]	Benefits Management Framework [168]	NASSS^a^ Framework [169]
Emphasis	Integration of a service. Continuity of care.	Benefits to health system. Service-level analysis.	Multilayered factors impacting adoption and spread of health technology.
Elements	Four patient-centric metrics: Resourcing and capabilities. Usability. Interoperability. Impact on end users.	Five workstreams: Customer and market insights. Behavioral economics. Data analytics. Impact evaluation. Health economic evaluation.	Seven broad factors: Condition: evaluates the suitability of technology for target health condition. Technology: specific aspects comprising suitability and usability. Value proposition for developer and end users. Adopters: maps agents in the adoption ecosystem. Organization: evaluates cultural factors impacting adoption or spread. Wider system: considers sociopolitical factors. Embedding and adaptation: a step intended for iteration, scoping, and promoting a culture of innovation.
Strengths	Evidence based on review of 28 articles. Aspires to digital health system responsiveness rather than mere adoption. Conscious of sociopolitical contexts.	Anticipates data for real-world analysis. Feasibility for real-world evaluation. Successfully piloted. Aimed at scalability	Prospective and anticipates potential hurdles to be overcome. Iterative and allows refinement of cultural nuances.
Weaknesses	Retrospective. Ignores other levers in the health system, such as health financing.	Retrospective. May overlook negative externalities. Service-focused and adoption-oriented rather than system-oriented. Neglects sociopolitical factors that are pivotal to system-wide adoption.	Service-focused. Places emphasis on guaranteeing adoption, not refining outcomes.

a NASSS: nonadoption, abandonment, scale-up, spread, and sustainability framework.

Similarly, health systems frameworks are individually limited: “Flagship Framework” [170] applies ethical, political, and policy cycle analyses, aiming at system-level diagnostics and strategic solutions, but underappreciates operational functions—the sort that AI addresses. By contrast, “Building Blocks Framework” [171] informs strategic decisions by policymakers and analysts, and operational decisions by program managers. However, it overlooks the dynamics of reforms, as could occur with AI’s adoption. An integrated health system framework—developed by Hsiao and Sparkes [172] and reframed by Sparkes et al [173]—unifies and improves on “Building Blocks Framework” [171] and “Flagship Framework” [170] (Table 4).

As illustrated in Figure 6, the integrated health systems framework considers ethical and political factors, and leadership capacities—which filter into public sector decisions (“control knobs”)—and links these to implications for health systems. These are then evaluated for system-level impacts (eg, efficiency and cost) and individual outcomes (eg, quality, access, health status) (Table 4). Such systems analysis could interrogate assumptions in economic models of AI, enabling determination of where efficiency gains accrue within the health system and potentially mitigate harm (Figure 6). In LICs such as South Sudan, analysis would proceed from a priori ethical consideration and prioritization—such as using AI in advancing equitable, efficient, and effective health care. Hereafter, control knobs such as health financing considerations will be tempered by political decisions around health financing goals and the power of associated technology firms, as well as regulatory capabilities to operationalize acquired technology. These macro-organizational factors have direct influence on the structure of out-of-pocket costs and, therefore, demand for health services. These indirectly influence outcomes such as the quality and efficiency of care and cost management in the health system. An intermediate moderating consideration between macro-organizational factors and outcomes is building blocks. These encompass factors such as drug supplies (as illustrated in Figure 5), information systems, and service delivery, which could leverage AI for better targeting of services.

The foregoing operationalization suggests that the integrated health systems evaluation framework could anticipate system-level impacts of AI. Efficiency gains from AI, for instance, would be filtered through a priori considerations—encompassing ethics, regulatory and financing capabilities, and human resources within health systems. Consequently, potential resource shunting from foundational health systems investment could render procurement of AI unethical, inefficient, and ungovernable for South Sudan. Instead, a sequenced approach prioritizing digital infrastructure and essentials of pharmacovigilance would be determined appropriate course of action. The current limitations of digital evaluation frameworks and insufficiency of individual health system evaluation frameworks could undermine AI global governance, especially as aggressive marketing may confound institutional weakness and result in misaligned health system investments in poor settings.

**Table 4.** Individual health systems evaluation frameworks are insufficient for AI.

Framework	WHO’s^a^ Building Blocks Framework [171]	World Bank’s Flagship Framework [170,172]	Integrated Health Systems Framework [172,173]
Emphasis	Supply side of health care, with intent on improvement, restitution, or maintenance of health.	National health systems as a vehicle for better health status, public satisfaction, and financial risk protection.	Unifies the strengths of the WHO framework and World Bank framework.
Elements	Examines all stakeholders and institutions of production in health care: Service delivery Health workforce Information Medical products and technology Health financing Leadership and governance	Control knobs: Financing Payment Organization Regulation Persuasion	Sequential consideration of enablers or barriers in the policy decision process: Ethical and political considerations. Leadership capacity. Links antecedent decisions to health system outcomes.
Target	Individual health outcomes. Efficiency Protection against financial risk	Access Equity Quality of care Efficiency	System level: efficiency and cost. Patient level: quality, access, health status, satisfaction.
Strengths	Patient-centric goals. Identifies broad areas of intervention	Outright consideration for ethical and political contexts of health reform agenda. Identifies actionable areas for policy interventions.	System-wide consideration Incorporates effect of antecedent decisions on public health outcomes. Identifies and links actionable interventions to health outcomes.
Weaknesses	Does not delineate how policymakers might influence outcomes. Lacks dynamism. Negates potential synergies among building blocks. Not amenable to iterative adoption. Not specific to digital health.	Discounts operational aspects of health systems. May underutilize program managers who often implement reforms in health systems. Not specific to digital health	Not specific to digital health

a WHO: World Health Organization.

**Figure 6.**
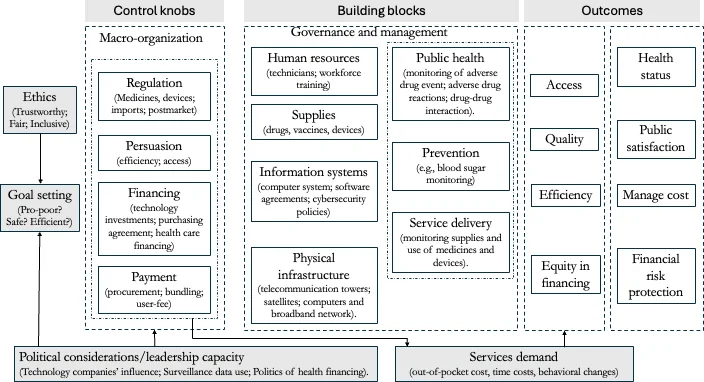
An integrated health systems evaluation framework is necessary for appraisal of AI. Source: adapted from Hsiao and Sparkes [172] and Sparkes et al [173].

## Regulating AI in Pharmacovigilance Demands Global Integration

Few countries in Africa are performing at maturity level 3 for medicines and vaccines regulation [174,175]. This implies regulatory stability and integration in less than 1% of African countries [176]. These include Ghana’s Food and Drug Authority, Tanzania’s Medical Devices Authority, Nigeria’s National Agency for Food and Drug Administration, the Egyptian Drug Authority, and Ethiopian Food and Drug Authority [175,177].

This incapacitation is worse for conflict-affected states like South Sudan, where the majority is impoverished [178] and tropical diseases such as onchocerciasis [179,180] and malaria [181] are prevalent. These conditions suggest it could benefit from innovations that circumvent geographical divides and infrastructure deficits [182]. Low regulatory capacity in South Sudan contributes to poor medicines regulation [183,184]. Nonetheless, it joined PIDM in 2024 [60], and its evolving technological landscape potentiates digitalization (Textbox 1). Digital health applications in South Sudan have aimed at efficiency in primary care [185] and distribution of mosquito nets [186]. However, these could extend to other services [23] and integrate AI. The improved access to internet that is afforded by Starlink for settings with limited infrastructure [187] improves the feasibility for South Sudan. Yet, an unguided procurement of AI in such settings risks “technological solutionism,” which could debase foundational health system investments.

South Sudan’s experience with Remote Access Community Hotspot for Education & Learning demonstrated the significance of relevant skills [188]. Similarly, efficiency gains from digitalization were demonstrated in its experience with Health Pooled Fund Quality-of-Care App, while underscoring the significance of suitable skills in technology adoption [189,190]. Allowing for suitable funding facility [191], programmed adoption [192], and network security constraints within the prevailing digital system [193-195], South Sudan’s experience also highlights the benefits of global integration through which cloud-based storage—which could be housed outside the sovereign jurisdiction and leveraged to support efficient and real-time analysis in regions with limited infrastructure [195-197]. Moreover, the disproportionate influence of global health funding agencies in settings such as South Sudan [198] presents the opportunity for humanitarian services to enhance AI-related health outcomes, while also underscoring that a poorly programmed adoption of AI could misallocate foundational health system investments. As identified in the first report of the UN’s Independent International Scientific Panel on Artificial Intelligence, limited capacity in LICs and skewed data used in LLMs would undermine effective global governance of AI [199]. Relegation of these low-income contexts within a “good AI society” [132] could impoverish global adoption of AI in pharmacovigilance.

Textbox 1. **A vignette on South Sudan.**

Declining cost of mobile phones and increased availability of internet—including connectivity via Starlink [187]—improve the feasibility of digital health. However, digital health services have yet to be fully operationalized in South Sudan, with constraints including scanty electrification and connectivity. These have limited adoption of proven digitally enabled models, such as the Aravind’s eyecare model [23] and simulation-based medical education [188]. Improved access to requisite technology and the evolving regulatory landscape [189] would potentiate AI and mobile health platforms.

In 2019, for instance, South Sudan operationalized the Health Pooled Fund Quality-of-Care Application (HPF QoC App) [190]. This provided a means to streamlining facility-level data entry for quality assessment of the Health Pooled Fund (HPF). The HPF concentrated multistakeholder financial resources for health care delivery in South Sudan [66,191], and performance evaluation had been slowed by inefficiencies and inaccuracies in data entry in health facilities. Deployment of the HPF QoC App is a test case for the feasibility of data-driven and mobile-based digital health platform [190]. Its adoption followed the prototypical diffusion of innovation [192] and had 39% early adopters (July-September 2019), and rapidly gained usage among 92.2% of facilities within 6 quarters (January-March 2021) [190].

Adoption of the HPF QoC App benefited from Training of Trainers between May and June 2019, ahead of implementation [190]. So, skills gaps would constrain scaling, an observation also made for an e-learning initiative on pharmaceutical management [65] and simulation-based medical education in South Sudan [188]. In the former, barriers included political fragility, technology, and language [65]. In the latter, the country has instructive experience with Remote Access Community Hotspot for Education & Learning, a tutor-dependent digital learning platform which operates offline using a local area network [188]. Coupling these mobile-based apps with large language models could boost adjudication of quality assurance across the health system, including pharmacovigilance. Even though web-based storage was enlisted for HPF QoC App [193], it proved the concept within the limits of currently available technology.

The experience of South Sudan also suggested that cloud storage, which could be housed anywhere, improves the feasibility of mobile-based tools. Except for network security concerns [194,195], a cloud-based health information system improves efficiency and capacity for real-time analysis at the point of care [195-197]. These make it amenable to integration with mobile-based platforms for surveillance of pharmaceuticals at border points, warehouses, health facilities, or community pharmacies. Moreover, it could support registries for quality assurance. Private pharmacies are currently underdeveloped in South Sudan but, with complementary innovations, they could bolster system-wide capabilities [24].

Furthermore, improvements in governance and infrastructure investments would facilitate rapid domestication and operationalization of these innovations in South Sudan. Global health practitioners and humanitarian services could enhance favorable outcomes through priority setting [66,198] and an iterative approach to capacity-building.

## Conclusion

This viewpoint sought to interrogate three related questions on moral trade-offs with the introduction of AI into health systems, the power dynamics that shape adoption, and how AI may be leveraged for pharmacovigilance in LICs. The viewpoint has established that AI is beneficially deployable in pharmacovigilance. However, low capacity in LICs constrains adoption of AI and undermines the evolving global regulatory regime. Moreover, an integrated health systems evaluation framework is necessary for anticipating and mitigating potential harms.

The viewpoint highlights emerging divergence in global capabilities in pharmacovigilance as AI gets integrated into advanced health systems while LICs struggle with building essential components of pharmacovigilance. This compounds the ethical concerns around AI, which encompass epistemic issues such as inscrutability and exclusivity of models behind AI; normative concerns such as cultural devaluation and unfair outcomes that segregate against minority groups; and metaethical issues such as the dominance of technology firms and a trust deficit, which render AI less governable. These could compound the current power imbalance in the global health system.

However, a synthesis of current applications of AI in pharmacovigilance uncovers potential gains for LICs, provided there is suitable investment in digital infrastructure in accompaniment of foundational health system investments. These applications range from detection of ADEs and ADRs, to processing of safety reports, to prediction of side effects and drug toxicity, which could guide personalized care. Even in well-resourced settings, these remain limited by data quality and also constrained by underrepresentation of certain population groups in clinical trials. Therefore, effective global governance of AI would demand that these applications are matched to necessary investments while not debasing foundational health system priorities in LICs.

Consequently, a suitable evaluation framework is important for health system planners, regulators, and global institutions of AI governance. The urgency gains with the need to anticipate downstream effects of AI adoption in health systems, especially because the inscrutability of the associated algorithms undermines the effectiveness of human-in-the-loop as a guardrail. Through interrogation of currently dominant digital and health system evaluation frameworks, the viewpoint identifies the suitability of using an integrated health systems evaluation framework to sequence risk identification and mitigation across the breadth of technology adoption. This approach is cognizant of the significant power vested by AI in a programmer or technology firm, which blunts physicians’ capacity to mitigate risk at the point of care. It suggests that, at the point of decision for acquisition, there must be a priori consideration of the ethics that would obtain, the macro-organizational dynamics, and the anticipated governance and managerial needs for the desired individual and system-level outcomes.

Finally, a vignette on South Sudan contrasted a low-capacity context against the advanced setting of Australia, underscoring the importance of primary health system investments, digitalization, and the benefits of global integration. As has been already highlighted in the preliminary report of the UN panel on AI, global imbalance in digital infrastructure and unrepresentative data would impair effective governance of AI. The juxtaposition of these varied contexts in this viewpoint systematically presents considerations for global governance of pharmacovigilance as it evolves with the adoption of AI.

## Abbreviations

ADE: adverse drug event
ADR: adverse drug reaction
DFCA: Drug and Food Control Authority
LIC: low-income country
LLM: large language model
PIDM: Programme for International Drug Monitoring
WHO: World Health Organization
